# Recharging and rejuvenation of decontaminated N95 masks

**DOI:** 10.1063/5.0023940

**Published:** 2020-09-01

**Authors:** Emroj Hossain, Satyanu Bhadra, Harsh Jain, Soumen Das, Arnab Bhattacharya, Shankar Ghosh, Dov Levine

**Affiliations:** 1 Department of Condensed Matter Physics and Materials Science, Tata Institute of Fundamental Research, Mumbai 400005, India; 2 Department of Physics, Technion-IIT, 32000 Haifa, Israel

## Abstract

N95 respirators comprise a critical part of the personal protective equipment used by frontline health-care workers and are typically meant for one-time usage. However, the recent COVID-19 pandemic has resulted in a serious shortage of these masks leading to a worldwide effort to develop decontamination and re-use procedures. A major factor contributing to the filtration efficiency of N95 masks is the presence of an intermediate layer of charged polypropylene electret fibers that trap particles through electrostatic or electrophoretic effects. This charge can degrade when the mask is used. Moreover, simple decontamination procedures (e.g., use of alcohol) can degrade any remaining charge from the polypropylene, thus severely impacting the filtration efficiency post-decontamination. In this report, we summarize our results on the development of a simple laboratory setup allowing measurement of charge and filtration efficiency in N95 masks. In particular, we propose and show that it is possible to recharge the masks post-decontamination and recover filtration efficiency.

## INTRODUCTION

Face masks are our first line of defense against airborne particulate matter.^1,2^ In particular, N95^34^ respirators comprise a critical part of the personal protective equipment (PPE) used by frontline health-care workers as they provide a barrier for transmission of pathogen laden droplets that are ejected by coughing, sneezing, talking, or breathing by an infected person.^3–6^ The name designation N95 indicates that these masks can filter 0.3 *μ*m sized particles with 95% efficiency.^4^ N95 masks are meant for one-time usage for two reasons: (1) potential contamination and (2) rapid degradation of their filtration efficiency with use. However, the recent COVID-19 pandemic has resulted in a serious shortage of these masks, which has started an intensive search for methods, which would allow for multiple uses.

Most of the literature has dealt with various proposals for decontamination procedures, including careful use of dry and wet heat or exposure to hydrogen peroxide vapor, ozone, UV radiation, or alcohol.^5,7–14^ While each of these methods likely deactivates viruses, it seems to be common knowledge that such procedures adversely impact filtration efficiency and may even cause deterioration of the structural integrity of the mask.

Less attention has been focused on restoring the filtration efficiency of a mask once it has become degraded; this is the question we address in this work. In this paper, we propose a method, which, provided the mask has not been structurally compromised, can restore the filtration efficiency to out-of-box levels.

As with other filtration processes, N95 masks intercept foreign particles in different layers of the mask material. A particle can be captured either *mechanically,* if it encounters a mask fiber directly in its path, or *electrostatically,* if the mask material is such that it can attract and ensnare particles.^16^ On making contact with the surface of the fiber, adhesive forces, such as the van der Waals force, immobilize the particle on the surface of the fiber.^17^

Flow through a mask is usually thought to be laminar such that the flow would usually bend smoothly around an obstacle (fiber). If this is the case, mechanical capture of the particle on the surface of the fiber happens when a particle deviates from its streamline path, causing an impact with the mask material. This can happen for larger particles whose inertia is large enough to cause such a deviation from the streamline or for smaller particles whose Brownian diffusion is strong enough.^18^ For filters based on fibrous materials and operating at filtration velocities similar to those encountered in human breathing, the minimum filtration efficiency occurs for ≈0.3 *μ*m sized particles. At this scale, the filtration mechanism crosses over from a diffusion dominated regime to an inertia dominated regime.^19^

In addition to mechanical capture, N95 respirators employ an electrostatic mechanism to attract and intercept foreign particles (charged or uncharged). This happens when there are significant electric fields and electric field gradients in the mask material, which may occur when the fibers are charged.^20^ It is these electrostatic interactions that raise the filtration of N95 masks to the 95% level. Charged fibers can attract both inherently charged particles by Coulombic forces and neutral polar particles (such as tiny aqueous droplets) by dielectrophoretic forces that come from the interaction of polarized objects and electric field gradients.

In typical N95 masks, the electrostatic filtration is performed by a layer comprised of a non-woven melt-blown mesh of charged polypropylene fibers. Most of the pores in this mesh have a characteristic length scale of about 15 *μ*m, and about 90% of its space is void. This layer is held in place between two or more quasi-rigid layers that provide both support and mechanical filtration. Polypropylene is an *electret*, a dielectric material, which can hold a charge or possess a net microscopic dipole moment.^21^

Pure polypropylene is a non-polar polymer with a band gap of 8 eV. However, the presence of molecular level defects both chemical and physical in nature allows the formation of localized energy states that can trap charge.^21^ Moreover, its electrical polarization properties are often enhanced by introducing various additives such as magnesium stearate^22^ or BaTiO_3_,^23^ which are added to the polymer melt to increase the electret performance. Even then, the charge on the polypropylene fibers undergoes significant degradation when open to the surroundings, which is exacerbated by the warm humid environment created by respiration during use. Additionally, most decontamination methods remove all the charges from the charged layer, with a concomitant reduction in mask efficiency.

Thus, a key aspect of the performance of an electret-based mask is its ability to maintain its charge in a hot and humid atmosphere. Failing this, extended usage can only be obtained through a cycle of decontamination and recharging if this is possible. It follows that a simple procedure for electrically recharging a decontaminated mask without disassembling it would be very useful, especially if it does not rely on special-purpose equipment, which would not be readily available.

The standard methods for charging polymer fibers are corona discharge,^24^ photo-ionization induced by particle beams (gamma rays, x-rays, and electron beams),^25,26^ tribo-electrification,^27–29^ and liquid contact charging.^30^ These methods are not easily deployable in hospital conditions on preassembled masks. In this note, we propose a simple recharging method based on high electric fields and demonstrate its effectiveness.

Crucially, our method can be performed using readily available equipment and materials and so can be employed both in urban and rural settings.

## MASK FILTRATION TESTING SETUP

**FIG. 1.**
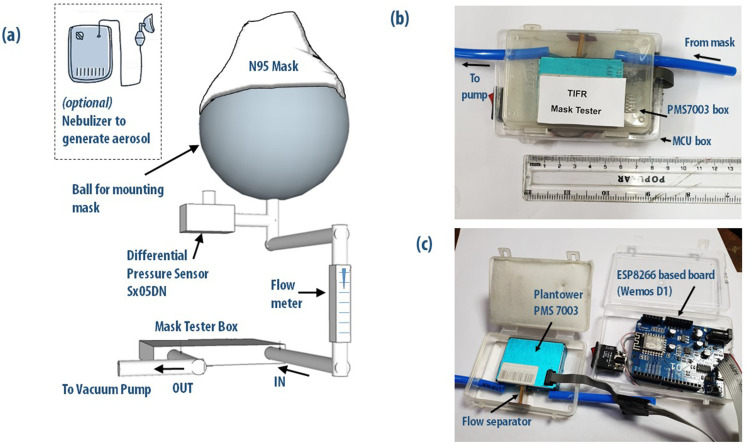
(a) Schematic diagram of the compact, low-cost mask tester developed in the lab. The mask is attached to a hard plastic ball simulating a human head, and air flow through the mask was affected by a small diaphragm pump. Particle counts were performed by using a Plantower PMS7003 air quality sensor chip, which was interfaced to an ESP8266 WiFi micro-controller unit. (b) View of the air quality sensor chip and control unit in small plastic boxes kept atop each other in a compact configuration. (c) Opened up view showing the PMS7003 chip and the ESP8266-based MCU board. The box edges and connector ports are hermetically sealed to ensure that the setup is airtight. Additional details of the experimental setup can be found in the GitHub repository^15^ or in the supplementary material.

Because of the COVID-19 pandemic, we did not have access to special-purpose mask filtration equipment, so we designed and constructed a rough apparatus to measure the efficiency of filtration of particulate matter using an air-quality monitor as a particle counter. The setup is shown in Fig. 1. A plastic ball serves as our proxy of the human face, on which we place the mask that we want to test. Air is sucked through the mask with a vacuum pump whose flow rate is controlled and monitored by using a flow meter. We use an oil-free diaphragm pump (HSV-1, High Speed Appliances, Mumbai) that provides a maximum flow of 30 lpm. The flow can be measured and controlled with a taper-tube flow meter. For most experiments, we used a flow of 10 lpm, similar to typical human breathing rates. This air is made to flow through a particle counting setup, which contains a Plantower PMS7003 sensor.^35^ The details of the experimental setup can be found in the GitHub repository^15^ or in the supplementary material.

While the particle sensor chips are optimized for 2.5 *μ*m particle measurements, the Plantower PMS7003 sensor also has a 0.3 *μ*m channel. The filtration efficiency (*η*) is determined from the ratio of 0.3 *μ*m particles per unit time detected with the mask attached (*N*_*mask*_) to that without the mask attached (*N*_*ambient*_) as

$$\eta =\left(1-\frac{N_{mask}}{N_{ambient}}\right)\times 100.$$

Our measurements are taken using a small diaphragm pump to suck air through a mask attached to a plastic ball at flow rates (∼10 lpm) of the order of physiological breathing rates. Much higher flow rates (∼80 lpm) are often used to certify N95 masks. To check for the dependence of the filtration efficiency on flow rates, we measured the filtration efficiency for flow rates between 3 lpm and 30 lpm and have found that the difference in the measured *η* is about one percent. The efficiency of our particle counter for smaller particles is of order 50%. Since *η* is related to the ratio of *N*_*mask*_ and *N*_*ambient*_, it is insensitive to the fact that not all the particles at 0.3 *μ*m are being counted. We have cross-checked the measurements obtained with the Plantower chip with a Lighthouse clean-room particle counter, and we found the measurements of *η* by both the devices to be consistent. For the ambient air to be filtered, we generated aerosols of normal saline solution (0.9%) by employing a standard medical nebulizer. These nebulizers produce a broad distribution of droplet sizes ranging from 100 nm to 10 *μ*m.^31^

**FIG. 2.**
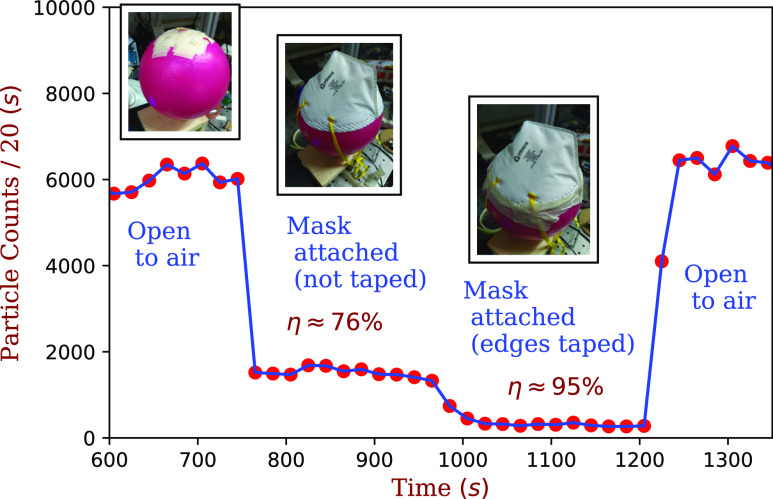
Filtration tests on a pristine Venus 4400 N95 mask. For the initial and final readings, no mask was attached, which serves as a baseline. When untaped, the seal between the mask and the ball is imperfect, and the filtration efficiency is 76% ± 1%. Upon taping the mask to the ball, we obtain ∼95% ± 1% filtration efficiency.

The fit of the mask to the plastic ball is imperfect, allowing air leakage from the sides. To obtain reproducible values, the mask edges were taped to the ball using paper masking tape. The filtration data, albeit employing a home-made testing apparatus, should be sufficient to make at least semi-quantitative comparisons between one mask and another and quantitative comparisons between the same mask in its charged and uncharged states. To give a sense of the measurement, the filtration data from a pristine N95 mask are shown in Fig. 2. When the mask is placed on the ball without taping the sides, its efficiency was 76% ± 1%. Upon taping the sides, the efficiency improved to 95% ± 1%. The reduction in the filtering efficiency due to poor fitting is a generic problem associated with the use of face masks.^1^

## CHARGE MEASUREMENT

**FIG. 3.**
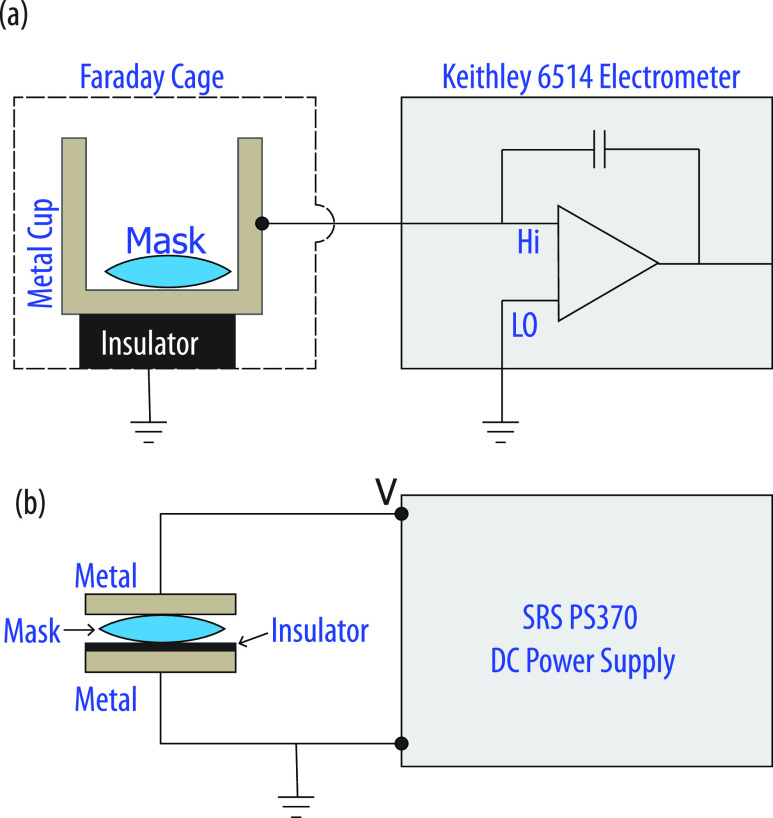
Schematic of the charge measurement setup (a) and mask recharging (b). In the mask recharging setup, a 100 *μ*m thick insulator [Polyethylene terephthalate (PET) plastic sheet] was inserted between the mask and the ground electrode. Under high field, the mask acts like an electrode on which the charges can be deposited. The insulator allows the mask to get charged because it prevents any current from flowing in the circuit.

As shown in Fig. 3(a), we used a Keithley 6514 electrometer to measure the charge, with the mask placed in a metal cup, which was electrically isolated from the ground by an insulating Teflon surface. The input of the electrometer is a three-lug triax connector, with the innermost wire (input high) being the charge sensing terminal. This charge sensing terminal of the electrometer was connected to the metal cup. In our experiments, we used the guard-off condition, i.e., the common (input low) and the chassis are grounded. Electrometers measure charge by transferring the charge from the point of measurement to the reference capacitor of the electrometer, and only free charge can be transferred. Therefore, since it does not account for any bound charge, our measurement likely underestimates the total charge on a mask and so should be regarded as giving a relative indication rather than a precise measurement of the total charge on the masks. This being said, there appears to be a *qualitative* correlation between measured charge and filtration efficiency, with masks with higher values of measured charge having higher filtration efficiency *η* (see Table I). The data of both N95 and surgical masks are tabulated in Table I. The surgical masks are different than the N95 masks in construction. Hence, comparisons between charge and filtration efficiency should be made between masks of the same type. Moreover, our charge measurement technique is not sensitive to the dipolar character of the electrets. Hence, quantitative calculation of correlation based on free charges cannot be estimated from this measurement alone.

**TABLE I.** Filtration efficiency and charge of masks tested. The surgical masks are different than the N95 masks in construction; thus, the comparisons between charge and filtration efficiency should be made between masks of the same type. The error in the charge measurement is mainly statistical in nature and comes from the uncertainty in the contact between the mask and the electrode. Note that the resolution of the charge measurement capability of the Keithley 6514 electrometer is few femto coulombs, which is orders of magnitude smaller than what is measured.

Brand	Mask	Filtration	Charge
name	type	efficiency (%)	(nC)
O&M Halyard 4627	N95	98 ± 1	9 ± 0.5
Venus-V4420	N95	96 ± 1	1 ± 0.5
Primeware Magnum	N95	95 ± 1	1 ± 0.5
K95	N95	95 ± 1	1 ± 0.5
Magnum Viroguard	Surgical	98 ± 1	8 ± 0.5
FFP1 3-ply			
Magnum SMS 3-ply	Surgical	79 ± 1	2.9 ± 0.5
Magnum 3ply	Surgical	65 ± 1	1.3 ± 0.5

## RECHARGING

The masks were recharged by sandwiching them between two metal plate electrodes, which were connected to the high and the low output terminals of a SRS PS370 power supply. The low output terminal was grounded, and a suitable voltage of positive or negative polarity was applied from the high output terminal of the source meter; Fig. 3(b) sketches the recharging setup.

Our recharging method exploits the nonlinear conductivity of electrets, in particular, polypropylene, as a function of the applied electric field. The electrical conductivity of polypropylene is dominated by hopping.^32,33^ Thus, at high fields, the conductivity of polypropylene is high, which makes the introduction of excess charges into the material possible by connecting it to a charge source.

**FIG. 4.**
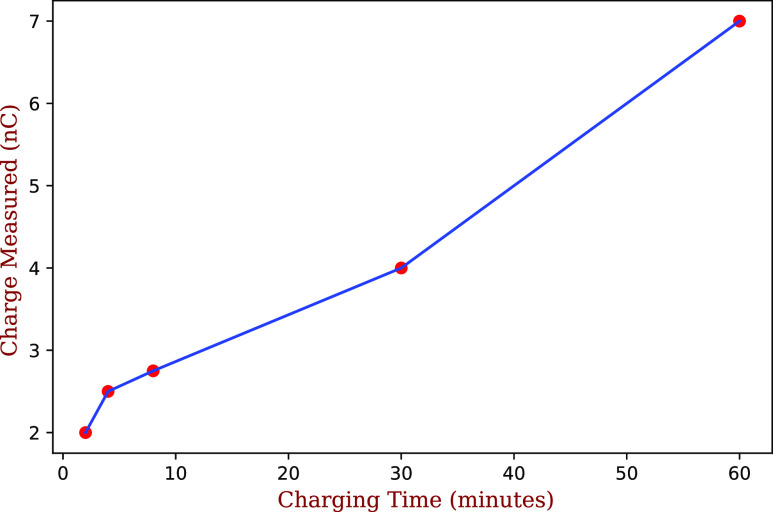
Charge accumulated on an O&M Halyard 4627 mask as a function of charging time at 1000 V. The charge on the pristine mask was ∼8 nC.

When the charge source is switched off, the applied electric field becomes zero, and conductivity of the polypropylene drops effectively to zero. As a result, the added charge carriers become immobile, and the material remains charged. We find that the total charge deposited on the masks depends strongly on the charging time, as seen in Fig. 4, which shows the result of different charging times on a N95 mask, with the pristine value almost reattained after a 60 min charge at 1000 V.

## FILTRATION EFFICIENCY OF RECHARGED MASKS

In the section titled Recharging, we demonstrated that the application of a relatively high voltage recharges the masks. Of course, the important test is whether this recharging translates into improved efficiency in the filtration of fine particles. To assess this, we first obtained a baseline measurement for the filtration efficiency of new unused masks (the masks were not individually vacuum sealed). We then performed typical sanitization protocols, during which the masks typically lost most of their charge (we emphasize again that we are primarily measuring the free charge) and measured the filtration efficiency of the discharged masks. We then recharged the masks and measured their filtration efficiency. The effect of different sanitization protocols and recharging on the filtration efficiency of the masks is tabulated in Table II.

**TABLE II.** The drop in the filtration efficiency in N95 masks due to different protocols of decontamination and the recovery of filtration by recharging the masks. For the KN95 masks, we used 2000 V, while for the Venus and Magnum N95masks, we used 1000 V^a^. For decontamination by ethanol, a new KN95/Magnum mask was soaked in ethanol and then dried. In the boiling method, the mask was immersed in boiling water for 1 h and then dried. For the washing machine method, the mask was laundered in a regular washing machine in a standard 40 °C, 84 min cycle, wash/rinse/spin dry cycle. For the steam method, the Venus mask was exposed to steam for 5 min on each side. For all these protocols, we started with a new N95mask.

Sanitization method	Filtration efficiency (*η*)	
(mask brand)	Before recharge (%)	After recharge (%)
Ethanol KN95	90 ± 1	96 ± 1
Boiling water KN95	74 ± 1	86 ± 1
Washing machine KN95	75 ± 1	95 ± 1
Steam exposed	77 ± 1	86 ± 1
(Venus V-4420N95)		
Ethanol (Magnum N95)	50 ± 1	86 ± 1

^a^ We observed an increase in the efficiency by few percentages on increasing the recharging voltage from 1000 V to 2000 V. However, beyond 2000 V, a further increase in the applied voltage did not alter the efficiency of the mask appreciably.

**FIG. 5.**
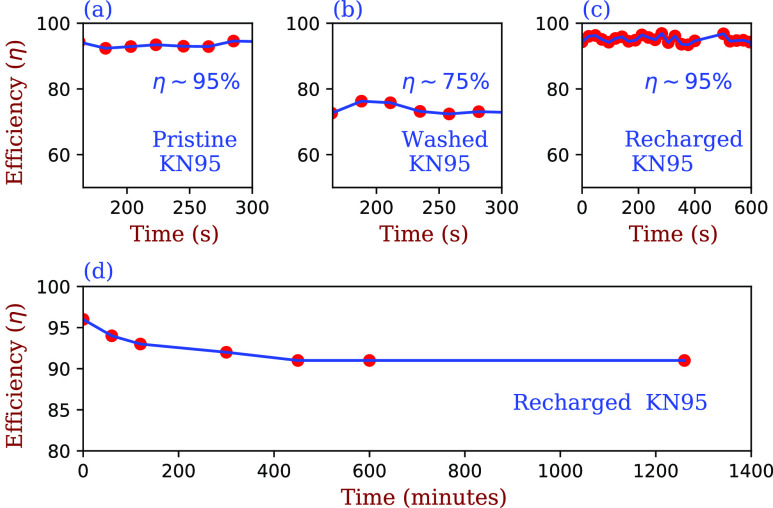
Top panel: (a) Comparison of the filtration efficiency of a new KN95 mask (95% ± 1%), (b) the same mask after washing and drying (75% ± 1%), and (c) the same mask after recharging for 60 min (95% ± 1%). The filtrations measured are for 0.3 *μ*m sized particles. The top rightmost panel of the figure shows that the filtering efficiency *η* is unchanged over a time span of 10 min. Bottom panel: (d) Decay of the efficiency of the recharged mask over the course of a day.

Representative data of the filtration efficiency of various masks after decontamination and recharging are given in the top panels of Fig. 5, where we start with a new KN95 mask whose out-of-box filtration efficiency was measured to be 95% ± 1% [see Fig. 5(a)]. The mask was then washed at ∼40 °C in a conventional washing machine with detergent. Such treatment would be expected to dissolve the lipid layer of the SARS-CoV-2 virus, which causes COVID-19. The mask was then air dried, and its efficiency was measured to be 75% ± 1% [see Fig. 5(b)]. The mask was then recharged for 60 min using the method of Fig. 3(b), following which its filtration efficiency was measured to be 95% [see Fig. 5(c)]. We then repeated this protocol and found that the filtration efficiency reattained 95% ± 1%. Figure 5(d) shows that the filtration efficiency of an exposed mask degrades only slightly, from ∼95% ± 1% to ∼92% ± 1%, over the course of one day. This suggests that the use of sterilization procedures, which do not cause structural damage to a mask coupled with our recharging protocol, will produce a respirator, which may be used multiple times with no sacrifice in filtration efficiency.

We have verified that the recharging method works on a variety of N95 respirators and that the filtration efficiency of degraded masks can be improved by charging, if not to brand-new efficiency. This suggests that by using this method, we should be able to determine the effect of various disinfection protocols on the structural integrity of different brands of mask. In this context, we note that a given sensitization method may affect different brands of masks very differently, as seen in Table II.

## *IN SITU* APPLICATION OF AN ELECTRIC FIELD KEEPS THE ELECTRET FILTER RECHARGED

Today, N95 masks are being worn by health-care workers for extended periods of time. This gives rise to very humid conditions. Humidity is detrimental to electrostatics. Thus, during use, all electrostatics-based masks slowly lose their efficiency. A solution that can help replenish the lost charge on the masks in real time would be desirable. In this section, we provide a proof-of-concept method of keeping the masks charged, which comes as a logical extension of our recharging method.

**FIG. 6.**
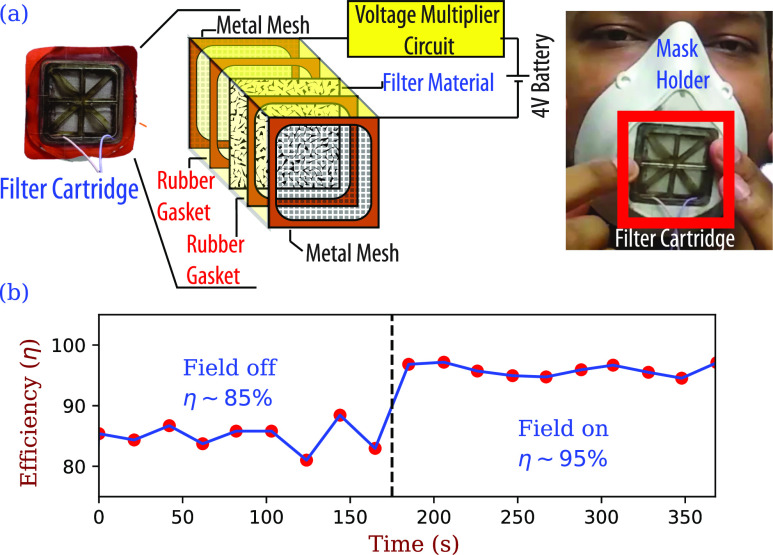
(a) Schematic representation of the *in situ* continuously charged mask, whose cartridge fits onto a 3D printed housing. (b) Upon applying a field, the efficiency of the cartridge improves from 85% ± 1% to about 95% ± 1%. Filtration measured for 0. 3 *μ*m sized particles.

We tested a technique by which the filter material maintains its charge and thus its filtration efficiency. We do this by applying a high electric field in a current limited condition [very low current (few *μ*A), so no risk of discharge or shocking] to the material *in situ*. Figure 6 shows a schematic of the *in situ* setup: a layer of filtration material (polypropylene mesh) cut from a standard N95 mask including all its layers serves as the filtration medium. This filter is sandwiched between two porous metallic screens, which are connected to a 4 V battery whose voltage is multiplied to 2000 V using a standard voltage multiplier circuit. We use a rubber gasket on both sides of the mask material to provide electrical insulation. The efficacy of the method is indicated in Fig. 6, where the filtration efficiency in the absence of an electric field is 85% ± 1%, which rises to 95% ± 1% upon application of voltage. We have verified that as long as the voltage is applied, the filtration efficiency remains high.

Since the currents required are extremely small, a large battery is not required, and it is possible that a small compact and practical solution may be feasible.

## CONCLUSIONS

Since the loss of electrical charge from the polypropylene filter layer in N95 masks is known to impact the filtration efficiency, we investigated the possibility of mask recharging for a few commercially available N95 masks using a simple laboratory setup. Our results suggest that it is possible to recharge the masks post-sterilization and recover filtration efficiency. However, this is a promising development that merits further research as it may permit multiple or extended use in practical applications. In particular, this method may allow for N95 masks to be used for a considerably longer period of time than is the current norm, which can have a significant effect in hospitals where mask supply is insufficient. Additionally, we envisage that our method may find applications in a variety of air filtration contexts. We have focused in this paper on high efficiency respirators for use in preventing disease transmission, but we anticipate applications to heating, ventilation, and air conditioning and industrial filtration as well, where our recharging method would allow for the extended use of electrostatic filters, resulting in reduced cost and waste. Furthermore, our *in situ* field application makes possible high efficiency filtration with undiminished performance over time.

## SUPPLEMENTARY MATERIAL

In the supplementary material, we outline the design of the low-cost, compact, particle filtration efficiency test setup using a Plantower PMS 7003 particle concentration sensor air quality monitor chip and a ESP8266-based WiFi microcontroller that was used for the measurements of particle filtration efficiency reported in this work. All the construction details, diagrams, and source codes for the micro-controller and interface are available in the supplementary material.

## DATA AVAILABILITY

The data that support the findings of this study are available within the article. Additional drawings and interfacing codes of the mask tester can be found in the GitHub repository.^15^
